# Intensive antihypertensive treatment does not lower cerebral blood flow or cause orthostatic hypotension in frail older adults

**DOI:** 10.1007/s11357-024-01174-4

**Published:** 2024-05-09

**Authors:** Ralf W. J. Weijs, Bente M. de Roos, Dick H. J. Thijssen, Jurgen A. H. R. Claassen

**Affiliations:** 1 Department of Medical BioSciences, Radboud University Medical Center, Nijmegen, The Netherlands; 2 Department of Geriatric Medicine (696), Radboudumc Alzheimer Center, Donders Institute for Brain, Cognition and Behaviour, Radboud University Medical Center, Geert Grooteplein Zuid 10, 6525 GA, P.O. Box 9101, 6500 HB Nijmegen, The Netherlands; 3 Research Institute for Sport and Exercise Sciences, Liverpool John Moores University, Liverpool, UK; 4 Department of Cardiovascular Sciences, University of Leicester, Leicester, UK

**Keywords:** Frailty, Geriatrics, Longevity, Orthostatic intolerance, Primary health care, Brain blood flow

## Abstract

This study aimed to examine the effects of intensive antihypertensive treatment (AHT), i.e., systolic blood pressure target ≤ 140 mmHg, on cerebral blood flow, cerebral autoregulation, and orthostatic hypotension, in a representative population of frail older adults. Fourteen frail hypertensive patients (six females; age 80.3 ± 5.2 years; Clinical Frailty Scale 4–7; unattended SBP ≥ 150 mmHg) underwent measurements before and after a median 7-week AHT targeting SBP ≤ 140 mmHg. Transcranial Doppler measurements of middle cerebral artery velocity (MCAv), reflecting changes in cerebral blood flow (CBF), were combined with finger plethysmography recordings of continuous BP. Transfer function analysis assessed cerebral autoregulation (CA). ANCOVA analysed AHT-induced changes in CBF and CA and evaluated non-inferiority of the relative change in CBF (margin: -10%; covariates: pre-AHT values and AHT-induced relative mean BP change). McNemar-tests analysed whether the prevalence of OH and initial OH, assessed by sit/supine-to-stand challenges, increased with AHT. Unattended mean arterial pressure decreased by 15 mmHg following AHT. Ten (71%) participants had good quality TCD assessments. Non-inferiority was confirmed for the relative change in MCAv (95%CI: -2.7, 30.4). CA remained normal following AHT (*P* > 0.05), and the prevalence of OH and initial OH did not increase (*P* ≥ 0.655). We found that AHT in frail, older patients does not reduce CBF, impair autoregulation, or increase (initial) OH prevalence. These observations may open doors for more intensive AHT targets upon individualized evaluation and monitoring of hypertensive frail patients.

Clinical Trial Registration: This study is registered at ClinicalTrials.gov (NCT05529147; September 1, 2022) and EudraCT (2022-001283-10; June 28, 2022).

**Supplementary Information:**

The online version contains supplementary material available at 10.1007/s11357-024-01174-4.

## Introduction

The global prevalence of hypertension has doubled in the past two decades [1], which is relevant since hypertension represents a leading modifiable risk factor for burden of disease [2]. Large RCTs have established that anti-hypertensive treatment (AHT) is beneficial, with substantial (~ 40%) risk reductions for cardiovascular events and mortality [3, 4]. Current treatment guidelines are moving towards lower SBP targets of 130–140 mmHg [5, 6]. Evidence supporting these guidelines mainly come from studies performed in non-frail individuals. For the rapidly increasing population of frail older patients, more conservative AHT is recommended [5–8]. These recommendations are based on the assumption that (intensive) AHT in frail older patients causes cerebral hypoperfusion or orthostatic hypotension (OH) [9, 10], leading to an increased risk for falls or dementia [11]. Importantly, the level of evidence supporting these recommendations is low, with few studies directly examining this relation.

The risk for cerebral hypoperfusion and OH with AHT in frail older individuals has long been linked to the widespread assumption that hypertension is a physiological, age-related adaptation to ensure sufficient CBF. However, this assumption has been questioned by recent studies. A recent meta-analysis found no negative effects of AHT on CBF in older hypertensive patients, including those with cognitive impairment [12]. Similarly, no evidence was found that AHT can impair cerebral autoregulation (CA) in older individuals [13]. Furthermore, in a large meta-analysis, intensive AHT did not increase the risk of OH [14, 15]. Together, these studies suggest that AHT does not cause cerebral hypoperfusion or OH in older, but mostly non-frail individuals. Evidence from studies in frail older adults however remains scarce. Therefore, the objective of this study was to directly examine the effects of intensive AHT, i.e., SBP ≤ 140 mmHg, on cerebral blood flow, cerebral autoregulation, and orthostatic hypotension, in a representative population of frail older adults.

## Methods

### Study design and participants

In this study, frail (Clinical Frailty Scale 4–7) older adults (age ≥ 70 years) with untreated or uncontrolled hypertension (unattended SBP ≥ 150 mmHg) were included for participation in this study. Detailed in- and exclusion criteria are listed in Table S1. Starting in September 2022, (records from) patients who visited the geriatric outpatient clinic between April 2022 and June 2023 were screened by a geriatrician for potential eligibility for participation in this explorative, single-arm intervention study. Potentially eligible patients received study information and, if they expressed interest in participation, were invited for a screening visit to confirm hypertension by an unattended BP measurement (at the clinic, or if preferred, at home). Before initiating or augmenting AHT, participants underwent baseline measurements in the research lab to assess cerebral hemodynamics, CA, and OH (lab visit 1). Subsequently, AHT was prescribed, and participants were visited two-weekly by a researcher to evaluate side effects or adverse events, and to perform home-based BP measurements. Once the treatment target (SBP ≤ 140 mmHg) was reached, lab measurements were repeated *in duplo* during follow-up on two separated days (lab visits 2 and 3 with 1–14 days in-between). A schematic overview of the study design with a participation flow-chart, is shown in Figure S1. The study was approved by the accredited local Medical Research Ethics Committee (METC Oost-Nederland, registration number NL80929.091.22) and conducted in accordance with the Declaration of Helsinki. All participants signed informed consent.

## Procedures

### Participant characteristics

Relevant clinically collected data, e.g. during the geriatric outpatient visit, were derived from the electronic patient dossiers (EPD), including general demographics, Clinical Frailty Scale (CFS), Montreal Cognitive Assessment (MoCA) scores, the TOPICS-Short Form questionnaire (TOPICS-SF, a validated Dutch PROM), (Instrumental) Activities of Daily Living (IADL/ADL) scores, medical history and medication use. In case information for the MoCA and/or TOPICS-SF questionnaire was missing from the patient records, the MoCA and/or TOPICS-SF was completed during the baseline lab visit.

### Blood pressure

BP measurements were performed using a Microlife WatchBP that automatically performs three assessments at the upper arm with 15-s rest intervals and stores an averaged output. Unattended BP measurements were performed as part of screening and during the three lab visits. During these assessments, participants were sitting quietly for ≥ 5 min, alone, to reduce the risk of ‘white coat hypertension’ and to prevent interaction with others that could impact BP levels. The two-weekly home-based BP measurements were similarly performed, but in the presence of a researcher who avoided moving or talking.

### Cerebral blood flow and autoregulation

During the three lab visits (one baseline and two follow-up), cerebrovascular measurements were performed under resting conditions with the patient seated on a chair for 5 min. Blood velocities in the left and right middle cerebral artery (MCA) were assessed using TCD (DWL Elektronische Systeme, Singen, Germany) by a trained ultrasonographer (RW). TCD is a non-invasive technique that applies ultrasound to track blood velocity changes in cerebral arteries, accessed through the transtemporal bone. Changes in mean bilateral blood velocity in the MCA (MCAv) represent changes in CBF under the assumption that the vessel diameter is constant, which has been confirmed under experimental conditions comparable to our study [16]. In addition, we measured beat-to-beat continuous BP using finger plethysmography (Finapres, Enschede, The Netherlands), three-lead electrocardiogram (Solar 8000 M, GE Healthcare, Milwaukee, WI, USA) and end-tidal carbon dioxide (EtCO_2_) (BIOPAC Systems, Goleta, CA, USA). All data were recorded continuously using a data acquisition system (Acqknowledge; BIOPAC Systems, Goleta, CA, USA).

### Orthostatic hypotension

To test for OH before and following AHT, participants performed a sit-to-stand and supine-to-stand postural change during the three lab visits. Following ≥ 5 min of seated/supine rest, patients were instructed to stand up to induce an orthostatic BP response, and to remain standing for 5.5 (sit-to-stand protocol) or 3.5 (supine-to-stand protocol) min while continuously recording heart rate (ECG) and beat-to-beat BP (finger plethysmography).

Sit-to-stand OH was defined as a drop of ≥ 15 mmHg in SBP or ≥ 7 in DBP after 1, 3 or 5 min of standing, while supine-to-stand OH was defined as a drop of ≥ 20 mmHg in SBP or ≥ 10 in DBP after 1 or 3 min of standing, relative to the resting value before standing up [17]. For both protocols, initial OH was defined in case the nadir, i.e. the lowest value upon standing up, was > 40 mmHg in SBP or > 20 in DBP lower compared to the resting value before standing up [18]. The supine-to-stand challenge more reliably tests for presence of OH and prognostic risk of falls compared to the sit-to-stand challenge [19].

### Anti-hypertensive treatment

In non-frail patients aged ≥ 65 years, European and Dutch guidelines for AHT advice a SBP target between 140–150 mmHg and, if well-tolerated, to consider a target between 130–139 mmHg [5, 20]. For frail older adults, guidelines let the treating physician decide the SBP target [5, 20]. For the purpose of this study, we have used the guidelines for non-frail patients aged ≥ 65 years, and used an unattended/home SBP target ≤ 140 mmHg. A detailed treatment protocol can be found in supplementary information A.

## Statistical analyses

Detailed information on data processing is described in supplementary information B. All statistical analyses were performed in IBM SPSS (version 27). Normal distribution of continuous variable data was checked visually. For descriptive data on participant characteristics at baseline, normally distributed data are presented as mean with standard deviation, and non-normally distributed data as median with interquartile range. Categorical data are presented as frequency number with percentage. Figures are used to show individual values from unattended/home BP measurements, resting hemodynamics and CA parameters, accompanied by means with 95% confidence intervals.

Follow-up values for continuous measures were calculated as the average of values derived during lab visits 2 and 3. ANCOVA analyses were performed to compare baseline and follow-up values of resting hemodynamics and CA parameters with a random intercept and with baseline values and the medication induced change in MAP as covariates. The level of statistical significance was set at *P* < 0.05. For our primary analysis, i.e., to test that AHT does not reduce CBF, these ANCOVA analyses were repeated for the relative change in MCAv from baseline to follow-up. Since we aimed to demonstrate absence of change, we adopted a non-inferiority design, with non-inferiority defined as the lower limit of the 95% confidence interval for the change in MCAv not exceeding the predefined margin of -10%. This margin was taken because 10% variation in CBF is expected under physiological conditions across a wide range of MAP, i.e. ~ 50–150 mmHg [13]. AHT-induced changes in MCAv exceeding twice this variation, i.e. > 20%, were carefully re-evaluated (e.g. to check for measurement error) to verify whether changes can be classified as non-physiological outliers. In case outliers were detected, ANCOVA analyses were repeated without individual data from these participants.

Individual BP data from sit-to-stand and supine-to-stand challenges are presented using spaghetti plots. The McNemar test was used to analyse whether paired proportions of initial OH and OH (i.e. yes/no based on both challenges) differed following AHT [21]. In addition, repeated measures ANOVA analyses were performed to examine whether SBP and DBP responses during sit-to-stand and supine-to-stand challenges were different between baseline and follow-up.

## Results

Fifteen hypertensive frail older adults were included in this study. One participant resigned from study participation before the initiation of AHT, leaving fourteen participants who completed study participation by undergoing baseline measurements and at least one session of follow-up measurements. Due to insufficient TCD signal quality, assessments of MCAv and CA were not performed in four (29%) of these participants (Figure S1). Detailed participant characteristics and baseline medication use are presented in Tables 1 and 2, respectively.

**Table 1:** Participant characteristics

	All (N = 14)	With TCD (n = 10)	Without TCD (n = 4)
Age, years	80.3 ± 5.2	80.1 ± 6.1	80.1 ± 2.1
Female sex, n (%)	6 (43)	2 (20)	4 (100)
Unattended BP during screening			
SBP, mmHg, n (%) DBP, mmHg, n (%) SBP 150–159 mmHg, n (%) SBP ≥ 160 mmHg, n (%)	164 ± 10 87 ± 11 6 8	163 ± 9 90 ± 11 4 6	167 ± 13 78 ± 6 2 2
Marital status			
Married, n (%) Widow(er), n (%) Divorced, n (%)	7 (50) 6 (43) 1 (7)	6 (60) 3 (30) 1 (10)	1 (25) 3 (75) 0 (0)
Living situation			
Independently together, n (%) Independently alone, n (%) Retirement home, n (%)	7 (50) 6 (43) 1 (7)	6 (60) 3 (30) 1 (10)	1 (25) 3 (75) 0 (0)
Education level [30]			
Low, n (%) Middle, n (%) High, n (%)	7 (50) 5 (36) 2 (14)	4 (40) 4 (40) 2 (20)	3 (75) 1 (25) 0 (0)
Smoking status			
Never smoked, n (%) Former smoker, n (%) Current smoker, n (%)	7 (50) 5 (36) 2 (14)	4 (40) 4 (40) 2 (20)	3 (75) 1 (25) 0 (0)
Height, cm	169 ± 10	172 ± 9	161 ± 9
Weight, kg	79.7 ± 18.4	84.0 ± 19.4	69.0 ± 11.0
Body mass index, kg/m^2^	26.6 (24.5–29.2)	26.6 (24.5–30.1)	26.7 ± 3.1
Estimated GFR, ml/min/1.73m^2^	70.5 ± 16.0	70.7 ± 16.0	70.3 ± 16.2
Arterial sodium, mmol/l	140 ± 2	140 ± 2	141 ± 2
Arterial potassium, mmol/l	4.1 ± 0.3	4.1 ± 0.3	4.2 ± 0.3
CFS 4:5:6:7, n (%)	7:4:3:0 (50:29:21:0)	5:3:2:0 (50:30:20:0)	2:1:1:0 (50:25:25:0)
Subjective health, score (1–10) Subjective quality of life, score (1–10)	6.6 ± 1.0 7.2 ± 1.5^a^	6.8 ± 1.0 7.3 ± 1.6^a^	6.3 ± 1.0 7.0 ± 1.4
ADL dependency score 0:1:2:3^b^, n (%) Instrumental ADL dependency score 0:1:2:3^b^, n (%)	7:5:2:0 (50:36:14:0) 6:1:7:0 (43:7:50:0)	6:3:1:0 (60:30:10:0) 5:1:4:0 (50:10:40:0)	1:2:1:0 (25:50:25:0) 1:0:3:0 (25:0:75:0)
MoCA, total score (0–30) MoCA, MIS score (0–15)	19.8 ± 3.8 8.1 ± 4.4	20.7 ± 3.1 7.8 ± 3.5	17.5 ± 5.1 9.0 ± 6.7
Subjective memory complaints, n (%) Clinical cognitive decline, n (%) Mild cognitive impairment, n (%) Dementia, n (%) Anxiety disorders, n (%) Depression, n (%) Asperger’s syndrome, n (%)	11 (79) 10 (71) 8 (57) 2 (14) 1 (7) 4 (29) 1 (7)	9 (90) 7 (70) 5 (50) 2 (20) 0 (0) 2 (20) 1 (10)	2 (50) 3 (75) 3 (75) 0 (0) 1 (25) 2 (50) 0 (0)
Angina, coronary heart disease or myocardial infarction, n (%) Atrial fibrillation/flutter, n (%) Cardiac valve disease, n (%) Heart failure, n (%) Hypertension, n (%) Peripheral vascular disease, n (%) Stroke/TIA, n (%) Small vessel disease, n (%)	5 (36) 3 (21) 4 (29) 1 (7) 14 (100) 2 (14) 1 (7) 1 (7)	3 (30) 2 (20) 2 (20) 0 (0) 10 (100) 2 (20) 0 (0) 1 (10)	2 (50) 1 (25) 2 (50) 1 (25) 4 (100) 0 (0) 1 (25) 0 (0)
Chronic kidney disease (estimated GFR < 60) Diabetes	3 (21) 3 (21)	2 (20) 1 (10)	1 (25) 2 (50)
Arthritis/arthrosis/osteoporosis Cancer within past 5 years Asthma / COPD	3 (21) 2 (14) 1 (7)	2 (20) 1 (10) 1 (10)	1 (25) 1 (25) 0 (0)
Visual/auditory impairment, n (%) Polyneuropathy, n (%) Balance problems, n (%) Dizziness, n (%) Fall within past year, n (%)	8 (57) 3 (21) 9 (64) 8 (57) 7 (50)	6 (60) 2 (20) 7 (70) 5 (50) 5 (50)	2 (50) 1 (25) 2 (50) 3 (75) 2 (50)

^a^1 missing value. ^b^Score categories: 0 = fully independent, 1 = moderately impaired, 2 = severely impaired, 3 = fully dependent [31]

ADL, activity of daily living; BP, blood pressure; DBP, diastolic blood pressure; COPD, chronic obstructive pulmonary disease; GFR, glomerular filtration rate; SBP, systolic blood pressure; TCD, transcranial Doppler; TIA, transient ischemic attack

**Table 2:** Medication use at baseline

Medication use, n (%)	All (N = 14)	With TCD (n = 10)	Without TCD (n = 4)
Polypharmacy (use of ≥ 5 prescribed medications)	11 (79)	7 (70)	4 (100)
Cardiovascular medication			
Antihypertensives Betablocker(s) ACE inhibitor(s) ARB CCB Thiazide diuretic(s) Spironolactone Statins Loop diuretic(s) Digoxin Nitrates Salicylates Anti-coagulants Platelet-inhibitor(s) DOAC	12 (86) 6 (43) 5 (36) 4 (29) 4 (29) 2 (14) 2 (14) 5 (36) 1 (7) 1 (7) 2 (14) 4 (29) 4 (29) 1 (7) 3 (21)	8 (80) 3 (30) 3 (30) 3 (30) 3 (30) 1 (10) 0 (0) 2 (20) 1 (10) 0 (0) 2 (20) 3 (30) 3 (30) 1 (10) 2 (20)	4 (100) 3 (75) 2 (50) 1 (25) 1 (25) 1 (25) 2 (50) 3 (75) 0 (0) 1 (10) 0 (0) 1 (10) 1 (10) 0 (0) 1 (10)
Diabetes medication			
Biguanides	2 (14)	0 (0)	0 (0)
Psychotropic medication			
Antidepressants Tri-/tetracyclic antidepressant(s) SSRI Benzodiazepines Antiepileptic Cannabidiol oil	4 (29) 3 (21) 1 (7) 1 (7) 1 (7) 1 (7)	2 (20) 1 (10) 1 (10) 0 (0) 1 (10) 1 (10)	2 (50) 2 (50) 0 (0) 1 (25) 0 (0) 0 (0)
Urological medication use			
Alfa blocker 5-alpha-reductase inhibitors Spasmolytics	1 (7) 1 (7) 3 (21)	1 (10) 1 (10) 3 (30)	0 (0) 0 (0) 0 (0)
Other medication use			
Bisphosphonates Proton pump inhibitors Vitamin/mineral supplements Inhalational parasympatholytics Laxatives Antispasmodics	1 (7) 6 (43) 8 (57) 1 (7) 4 (29) 1 (7)	1 (10) 3 (30) 7 (70) 1 (10) 3 (30) 0 (0)	0 (0) 3 (75) 1 (25) 0 (0) 1 (25) 1 (25)

ACE, angiotensin-converting enzyme; ARB, angiotensin II receptor blocker; CCB, calcium channel blocker; DOAC, direct oral anticoagulant; SSRI, selective serotonin reuptake inhibitor; TCD, transcranial Doppler

### Medication induced blood pressure changes

**Fig. 1:**
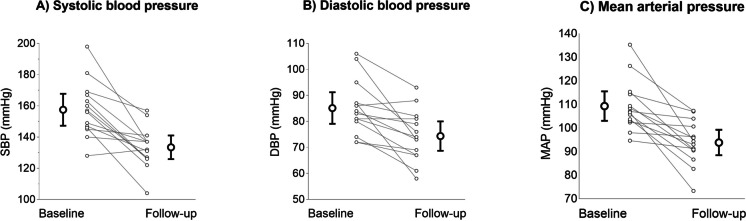
Means with 95% confidence intervals for unattended systolic (**A**) and diastolic (**B**) blood pressure, and mean arterial pressure (**C**), assessed during baseline and following antihypertensive treatment in the research lab, together with connected individual values

In thirteen participants (93%), AHT successfully reduced SBP to the treatment target of ≤ 140 mmHg (Figure S2A) across 6 ± 3 weeks of AHT. In one patient (7%), home and unattended SBP remained above target (i.e., 162 and 154 mmHg, respectively; Figure S2A and Fig. 1) despite 22 weeks of AHT, indicating uncontrolled hypertension. On average, unattended MAP was reduced across follow-up by 15 ± 12 mmHg (Fig. 1). All participants were included in the analyses. For an overview of medication used for AHT, see supplementary Table S2.

### Cerebral artery blood velocity and cerebral autoregulation

**Fig. 2:**
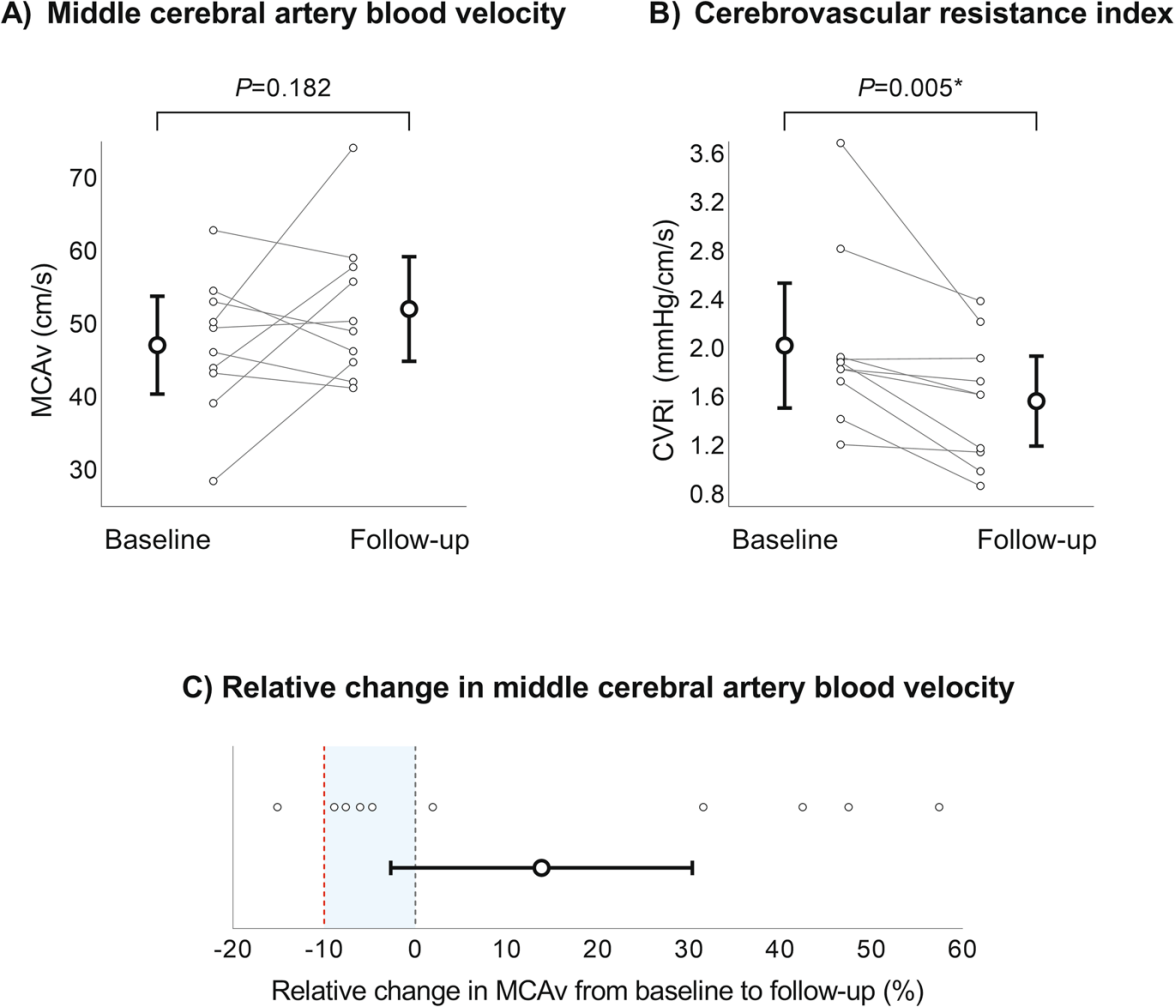
Figures A and B show means with 95% confidence intervals for middle cerebral artery blood velocity (**A**) and cerebrovascular resistance index (**B**) assessed during baseline and following antihypertensive treatment in the research lab, together with connected individual values and P-values derived from ANCOVA analyses. Figure C shows a forest plot for non-inferiority hypothesis testing based on the ANCOVA mean with 95% confidence interval with individual data. The red dashed vertical line represents the non-inferiority margin of -10%

ANCOVA analyses revealed no absolute change in MCAv across follow-up (Fig. 2A), whereas CVRi was reduced significantly (Fig. 2B). Regarding our primary outcome analysis, the change in MCAv following AHT (mean = 13.9%; 95%CI = -2.7, 30.4) did not cross the non-inferiority margin of -10% (Fig. 2C). At individual level, one participant exceeded the non-inferiority margin for MCAv (i.e., -15%). This participant also demonstrated the largest AHT-induced MAP reduction (i.e., -25 mmHg). We identified one non-physiological outlier, as insonation depths during TCD assessments at baseline and during follow-up were not comparable. Repeating the ANCOVA analysis without this individual reinforced non-inferiority (10.7%; 95%CI = -7.4, 28.7).

For CA, a statistically significant change was found only in one of eight parameters, i.e., transfer function gain in the low frequency domain (Figure S3). However, all other CA parameters over the low (Figure S3) and very low frequency domain (Figure S4) remained unchanged during AHT. Repeating these analyses without the statistical outlier did not alter the outcomes (Table S3).

### Orthostatic tolerance

**Fig. 3:**
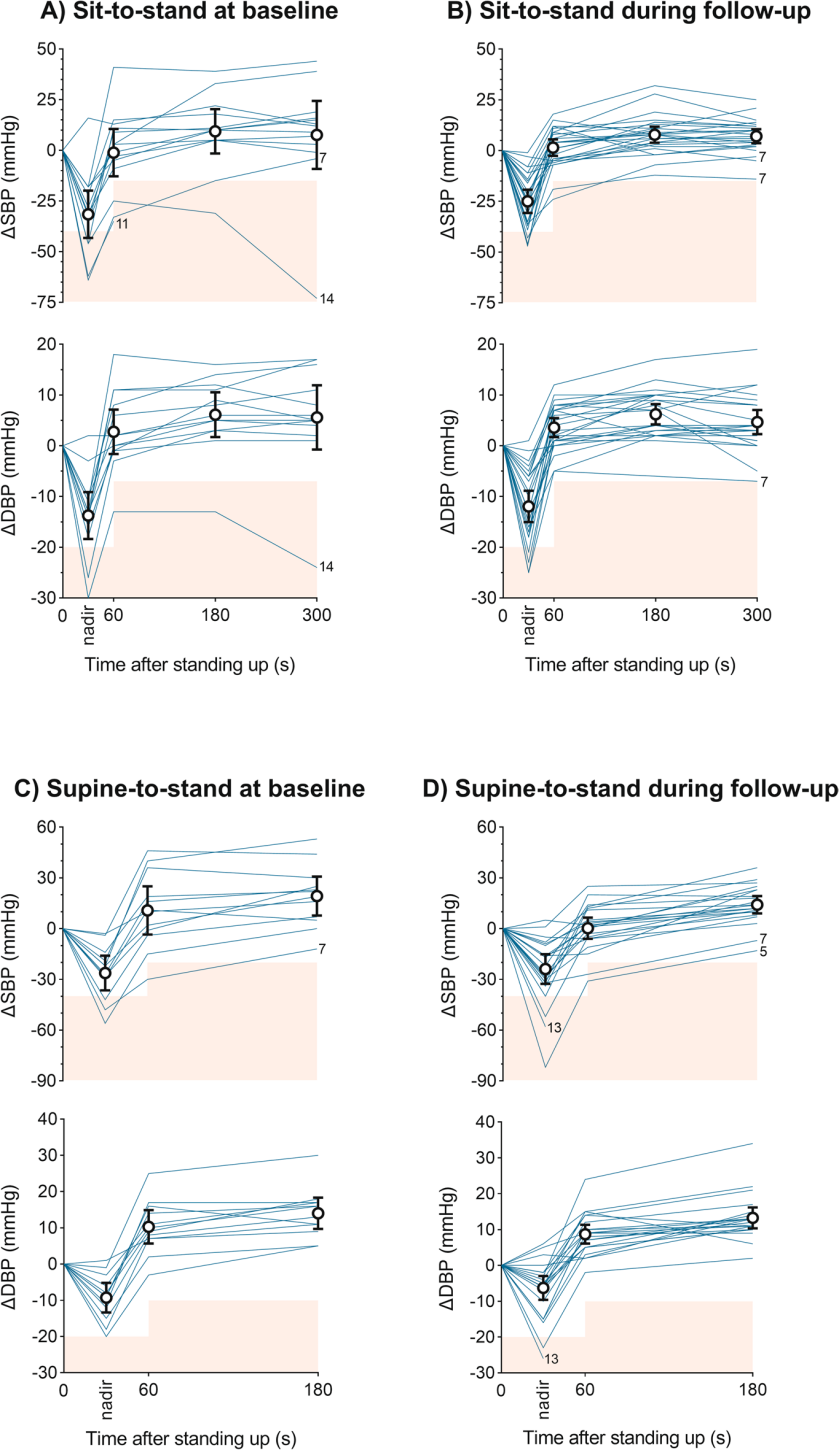
Spaghetti graphs showing individual trends for changes in systolic and diastolic blood pressure relative to seated resting values during the sit-to-stand challenge at baseline (**A**) and during follow-up (**B**), and relative to supine resting values during the supine-to-stand challenge at baseline (**C**) and during follow-up (**D**). Mean changes with 95% confidence intervals for the nadir and relevant timepoints are presented. The red areas indicate criteria for (initial) orthostatic hypotension. Spaghetti replicates pertaining to measurements during which criteria for orthostatic hypotension were met are labelled with the relevant participant number

All participants performed a sit-to-stand challenge at baseline and at least one sit-to-stand challenge at follow-up (Fig. 3A-B). The supine-to-stand challenge was performed at baseline and at least once during follow-up by 12 (86%) participants (Fig. 3C-D). During the sit-to-stand challenge, three individuals (21%) met the criteria for OH at baseline for SBP, which remained present for one (7%) during follow-up. This participant (#7) also met criteria for OH during the supine-to-stand challenge at baseline and during follow-up. None of the others met criteria for OH following AHT based on the sit-to-stand challenge. During the supine-to-stand challenge at baseline, only participant #7 met the criteria for OH. Two participants (#5 and #13) developed OH following AHT during the supine-to-stand challenge, with OH being symptomatic (dizziness) in one of them. Based on both challenges, prevalence of OH and initial OH did not change after AHT (OH: 21% to 21%, *P* = 1.000; initial OH: 36% to 43%, *P* = 0.655). Following AHT, absolute changes in SBP and DBP during sit-to-stand and supine-to-stand challenges did not differ from those at baseline (sit-to-stand: SBP, *P* = 0.846 and DBP, *P* = 0.898; supine-to-stand: SBP, *P* = 0.462 and DBP, *P* = 0.823).

## Discussion

We investigated the effect of intensive antihypertensive treatment targets for blood pressure in frail older adults on cerebral blood flow, cerebral autoregulation, and orthostatic hypotension. Intensive treatment lowered unattended MAP by 15 mmHg and met the SBP target of ≤ 140 mmHg in 13 out of 14 participants. Confirming our non-inferiority hypothesis, intensive treatment did not reduce CBF in these older, frail individuals. In line with this result, CA remained within normal ranges following AHT. Regarding postural hypotension, we found no increase in the prevalence of OH between baseline and AHT, and continuously measured BP responses during sit-to-stand or supine-to-stand postural changes showed no increase in initial OH. Altogether, our observations indicate that AHT with a treatment target of SBP ≤ 140 mmHg in this group of frail, older individuals did not cause cerebral hypoperfusion, impairment in CA, or higher prevalence of OH, which may have clinical relevance for antihypertensive treatment in this population.

Intensive AHT in these older, frail adults did not cause a reduction in CBF. This is in line with previous findings in non-frail older adults [12, 22]. For example, a recent meta-analysis, which includes the study by Lipsitz et al*.* [22], reported stable CBF following AHT-induced BP reductions in adults aged ≥ 50 years, while subgroup analysis even revealed increases in CBF in those aged > 70 years [12]. In addition, six studies included in this meta-analysis examined patients with mild cognitive impairment or dementia, and found no AHT-induced reduction in CBF.

It has been suggested that age-related hypertension represents a physiological adaptation, required to maintain CBF as older age leads to irreversible cerebrovascular stiffening [23, 24]. However, this theory has been challenged recently by evidence indicating that the cerebrovasculature has the ability to adapt [12]. Our observation that CBF is not reduced following AHT supports this notion, although we explored the effects of AHT on cerebrovascular parameters across a relatively short period. We did so because adverse effects of antihypertensive treatment are most frequently observed after initiation or augmentation of treatment. Nonetheless, long-term studies with repeated follow-up measurements are required to investigate whether these beneficial vascular adaptations persist to maintain sufficient CBF. This is especially relevant, as AHT primarily aims to provoke extended healthy longevity by reducing risk of morbidity and mortality by cardio- and cerebrovascular disease. Moreover, (intensive [3]) AHT reduces the risk of cognitive decline and dementia [25]. Given recent observations that reductions in CBF are associated with cognitive decline [26, 27], the apparent effect of AHT to increase CBF may contribute to the clinical potential of AHT for the prevention of dementia in frail individuals.

Previous work had suggested that AHT-induced CBF reductions in non-frail hypertensives may be related to a rightward shift in the CA curve [28]. Consequently, AHT would lead to impaired CA in case decreased BP levels fall below the lower limit of the rightward-shifted CA curve. Therefore, we examined AHT-induced changes in CA, and observed no CA impairment following AHT.

We then investigated the effects of treatment on the prevalence of both OH and initial OH, which, from a clinical perspective, represent a major concern as it causes dizziness and is associated with increased risk of falls. However, our results demonstrate no change in the prevalence of OH nor of initial OH. In addition to examining OH as a binary outcome using strict cut-off values, we also examined the absolute changes in BP, which reinforces our initial observation that AHT does not alter BP responses to orthostatic challenges in frail, older hypertensives. This is in line with previous evidence, primarily in non-frail individuals, concluding that intensive AHT reduces the risk of OH [14], while deprescribing AHT may increase the risk of OH [29]. Altogether, hypertension seems an important risk factor for OH, with lowering BP to ‘normal’ levels unlikely being a risk factor for OH, even in older, frail individuals.

### Strengths and limitations

Strengths of our study include the prospective, controlled design, and the inclusion of a well-defined and representative older population of frail individuals, recruited from a geriatric outpatient clinic. Frailty was rigorously assessed following a comprehensive geriatric assessment, and, in addition to the clinical frailty scores, is reflected in the number and types of comorbidity, the polypharmacy, cognitive impairment, and impairment in activities of daily living. Strengths also include the comprehensive, *in duplo* evaluation of main outcome parameters. However, some limitations must be considered. First, our statistical analyses regarding the prevalence of (initial) OH may be underpowered. However, our comprehensive protocol (including two orthostatic challenges, performed *in duplo*) and BP analysis on a continuous scale, strongly support our conclusion that AHT does not increase OH prevalence. Second, a limitation is that we could not measure potential vasodilatory effects of AHT on the MCA. The vasodilatory effect could have caused an increase in MCA diameter during follow-up. However, such an increase in MCA diameter, in the presence of preserved flow, would lead to a reduction in MCAv on follow-up. In contrast, we found no significant change in MCAv. Alternatively, if an increase in MCA diameter was present, this may have masked an increase in CBF on follow-up. Although we do not know what happened with the diameter, these observations further support our conclusion that AHT does not cause cerebral hypoperfusion in frail older adults. Third, our study has a small sample size, and would require replication in a larger study. Nonetheless, this study provides relevant evidence to support the rationale and ethical considerations regarding safety for such a study.

### Perspectives

Our observations suggest that successful blood pressure lowering following intensive AHT in frail older adults does not lead to cerebral hypoperfusion, impaired cerebral autoregulation, and/or increased prevalence of orthostatic intolerance. These observations argue against the assumption that AHT in frail individuals must be prevented because of the risk for cerebral hypoperfusion or orthostatic hypotension. Our results, therefore, strongly support future studies to examine the longer-term effects of intensive AHT in frail older adults, which is further supported by the clinical benefits of intensive AHT in the prevention of cardiovascular disease and dementia. Nonetheless, it remains important that AHT prescription is based on individualized comprehensive evaluation, especially pertaining to the heterogeneous responses observed in our study. As our study was not designed to specifically address mechanisms, we support future studies to better understand the (short- and long-term) potential risks and benefits of intensive AHT in frail older individuals, and to identify mechanisms explaining the preservation of cerebral perfusion upon AHT in this frail, older population.

### Supplementary Information

Below is the link to the electronic supplementary material.

Supplementary file1 (DOCX 3291 KB)

## Data Availability

A request for de-identified data can be submitted to the corresponding author. We are in the process of storing these data in a repository.
