# Supplementary material for: Intensive antihypertensive treatment does not lower cerebral blood flow or cause orthostatic hypotension in frail older adults
Source: GeroScience. 2024 May 9;46(5):4635–46. doi: 10.1007/s11357-024-01174-4 (PMC11335707; doi:10.1007/s11357-024-01174-4)
Supplement: Supplementary file 1 — Supplementary file1 (DOCX 3291 KB) [file 11357_2024_1174_MOESM1_ESM.docx]

**Supplemental material**

Information A: Treatment protocol

Individualized AHT was prescribed by a geriatrician (JC) after consideration of its safety for the participant, e.g. by taking renal and cardiovascular comorbidity and history into account. The medication classes used were angiotensin converting enzyme inhibitors, angiotensin II receptor blockers, calcium channel blockers, and/or thiazide diuretics. If indicated, betablockers and/or specific diuretics were additionally used for AHT. Rather than using a standard regimen, choices for drug class were based on comorbidity, co-pharmacy, and any allergies or contra-indications. In accordance with guidelines for older adults, the aim was to use the lowest dose for each class, with preference to add a second class rather than increase the dose of the first class when the BP target had not been reached. ^1^ In addition, the aim was to prescribe combination pills when possible. ^1^ Medication use was revised based on interim BP levels and possible side effects or adverse events monitored during the two-weekly home-based visits. To check medication adherence, participants were asked to record any deviations from the prescribed medication in a medication diary that was provided during the baseline lab visit.

*References*

1. Mancia Chairperson G, Kreutz Co-Chair R, Brunstrom M, et al. 2023 ESH Guidelines for the management of arterial hypertension The Task Force for the management of arterial hypertension of the European Society of Hypertension Endorsed by the European Renal Association (ERA) and the International Society of Hypertension (ISH). *J Hypertens*. Jun 21 2023;doi:10.1097/HJH.0000000000003480

Information B: Data processing

SBP and DBP values from unattended/home BP measurements were used to calculate MAP values. AHT-induced BP change was expressed as the average of MAP values from *in duplo* unattended BP assessments during follow-up (lab visits 2 and 3) minus the MAP value from the unattended BP measurement during baseline (lab visit 1). Participants were numbered ordered from the smallest to the largest MAP reduction.

According to international guidelines,^2,3^ transfer function analyses of data recordings from TCD assessments during the 5-minute resting condition (including control parameters) were performed to derive the mean MCAv, MAP, and EtCO_2_, and to derive parameters of cerebral autoregulation (CA). CA parameters include transfer function gain, normalized gain, phase, and coherence over the low (0.07-0.20 Hz) and very low (0.02-0.07 Hz) frequency domains, where CA is most active. Cerebrovascular resistance indices (CVRi) were calculated for each measurement (CVRi=MAP/MCAv). For each outcome parameter, single follow-up values were calculated by averaging the values from the *in duplo* follow-up assessments.

Continuous BP data recorded during the sit-to-stand and supine-to-stand challenges were automatically analysed using MATLAB scripts to calculate mean SBP and DBP values over 30-second periods for seated/supine rest (i.e. 40-10 seconds before standing up), and for the first, third, and fifth minute after standing up (i.e. 45-75/105-135/285-315 seconds after standing up, respectively). In addition, the SBP and DBP nadir, i.e., the lowest value upon standing up, were identified. For each measurement, all automatically yielded outcomes were visually verified based on graphs created by MATLAB. Ultimately, absolute changes in BP values from seated or supine resting values were calculated for relevant timepoints.

*References*

2. Panerai RB, Brassard P, Burma JS, et al. Transfer function analysis of dynamic cerebral autoregulation: A CARNet white paper 2022 update. *J Cereb Blood Flow Metab*. Jan 2023;43(1):3-25. doi:10.1177/0271678X221119760

3. Claassen JA, Meel-van den Abeelen AS, Simpson DM, Panerai RB, international Cerebral Autoregulation Research N. Transfer function analysis of dynamic cerebral autoregulation: A white paper from the International Cerebral Autoregulation Research Network. *J Cereb Blood Flow Metab*. Apr 2016;36(4):665-80. doi:10.1177/0271678X15626425

Table S1

| **Inclusion criteria** | **Exclusion criteria** |
| --- | --- |
| - Age ≥70 years; - Clinical Frailty Scale ≥4 and ≤7; - Diagnosis of untreated or uncontrolled hypertension, i.e. unattended office systolic blood pressure ≥150 mmHg; - Indication for AHT with the indication of primary or secondary prevention of vascular events, judged by the treating physician (geriatrician or primary care physician); - Able to understand and perform study related procedures. | - Unable to provide signed and dated informed consent form. - Mentally incompetent subjects as assessed by a physician; - Current participation in an interventional study targeting either blood pressure and/or cerebral blood flow; - Cardiovascular event within the past 3 months; - Estimated glomerular filtration rate <40 ml/min; - Known secondary cause of hypertension that causes safety concerns. |

Table S2: Medication initiated or augmented for antihypertensive treatment

| **Class(es) of used antihypertensive medication** | **All**  **(N=14)** |
| --- | --- |
| **ARB, n (%)** | 3 (21) |
| **CCB, n (%)** | 2 (14) |
| **TD, n (%)** | 2 (14) |
| **ARB+TD, n (%)** | 2 (14) |
| **ARB+CCB, n (%)** | 2 (14) |
| **ARB+CCB+TD, n (%)** | 1 (7) |
| **ACE+CCB+TD, n (%)** | 1 (7) |
| **ARB+CCB+BB+OD, n (%)** | 1 (7)^a^ |

Abbreviations: ACE, angiotensin-converting enzyme inhibitor(s); ARB, angiotensin II receptor blocker(s); TD, thiazide diuretic(s); OD, other diuretic(s).

^a^This participant did not reach the SBP target by AHT, and had a contraindication for using thiazide diuretics.

Table S3. Results from repeated analyses without statistical outlier (participant #10)

| **Parameter** | **Baseline (n=9)** | **Follow-up (n=9)** | **P-value** |
| --- | --- | --- | --- |
| **MCAv, cm/s** | 48.0±9.5 | 51.7±10.5 | 0.344 |
| **CVRi, mmHg/cm/s** | 2.06±0.75 | 1.63±0.50 | 0.007* |
| **Gain_LF_, cm/s/mmHg** | 0.63±0.31 | 0.84±0.21 | 0.029* |
| **nGain_LF_, %/mmHg** | 1.32±0.58 | 1.62±0.42 | 0.086 |
| **Phase_LF_, degrees** | 22.1±18.7 | 27.4±10.4 | 0.191 |
| **Coherence_LF_, units** | 0.59±0.27 | 0.65±0.25 | 0.464 |
| **Gain_VLF_, cm/s/mmHg** | 0.49±0.18^a^ | 0.61±0.16^b^ | 0.205 |
| **nGain_VLF_, %/mmHg** | 1.12±0.61^a^ | 1.15±0.27^b^ | 0.609 |
| **Phase_VLF_, degrees** | 55.5±33.0^a^ | 46.7±21.9^b^ | 0.511 |
| **Coherence_VLF_, units** | 0.54±0.20^a^ | 0.54±0.15^b^ | 0.224 |

^a^2 missing values. ^b^1 missing value.

Abbreviations: CVRi, cerebrovascular resistance index; LF, low frequency domain; MCAv, mean bilateral blood velocity in the middle cerebral artery; VLF, very low frequency domain.


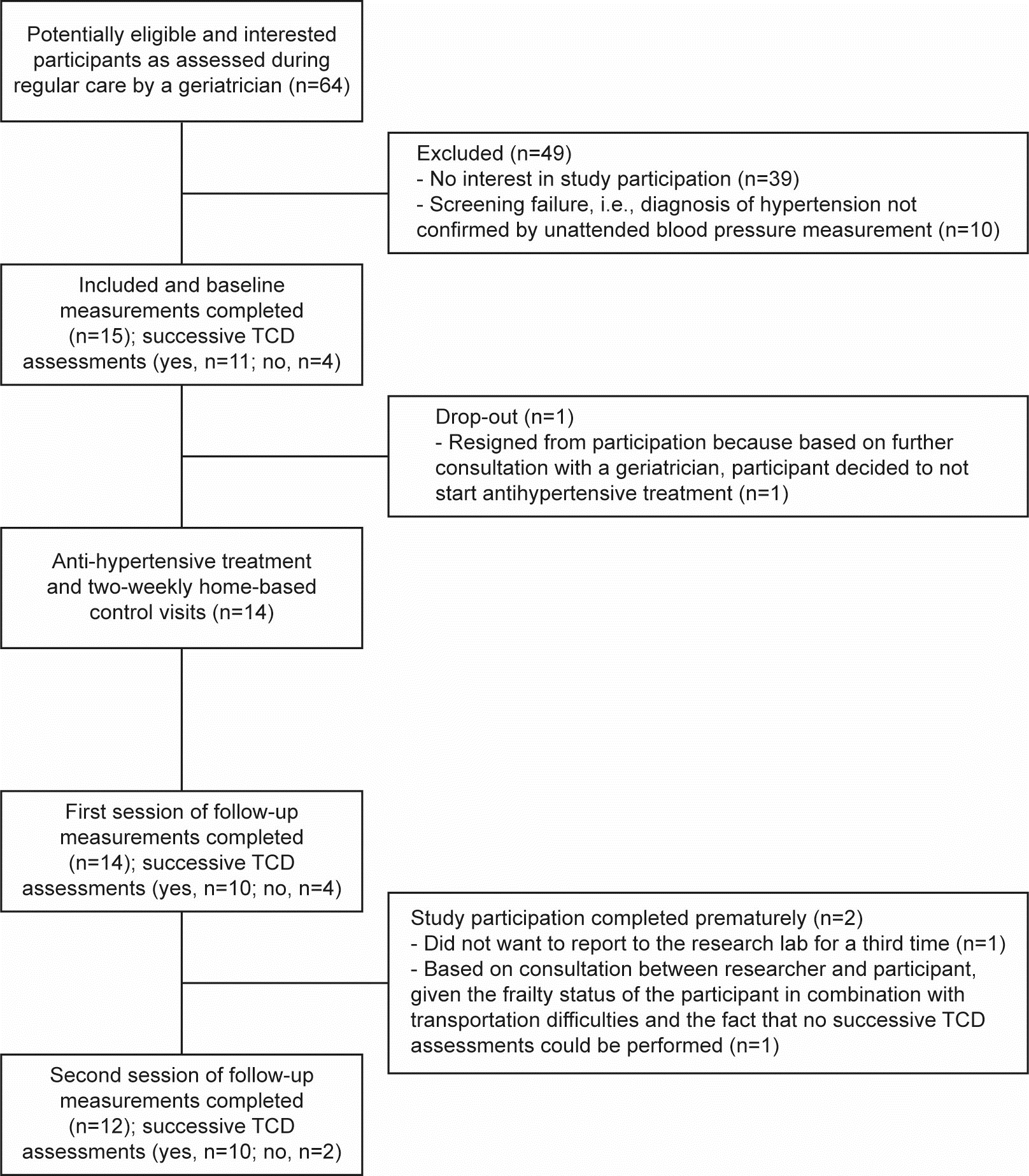


**Figure S1.** Schematic overview of the study design and flow of participants. Abbreviations: TCD, transcranial Doppler.


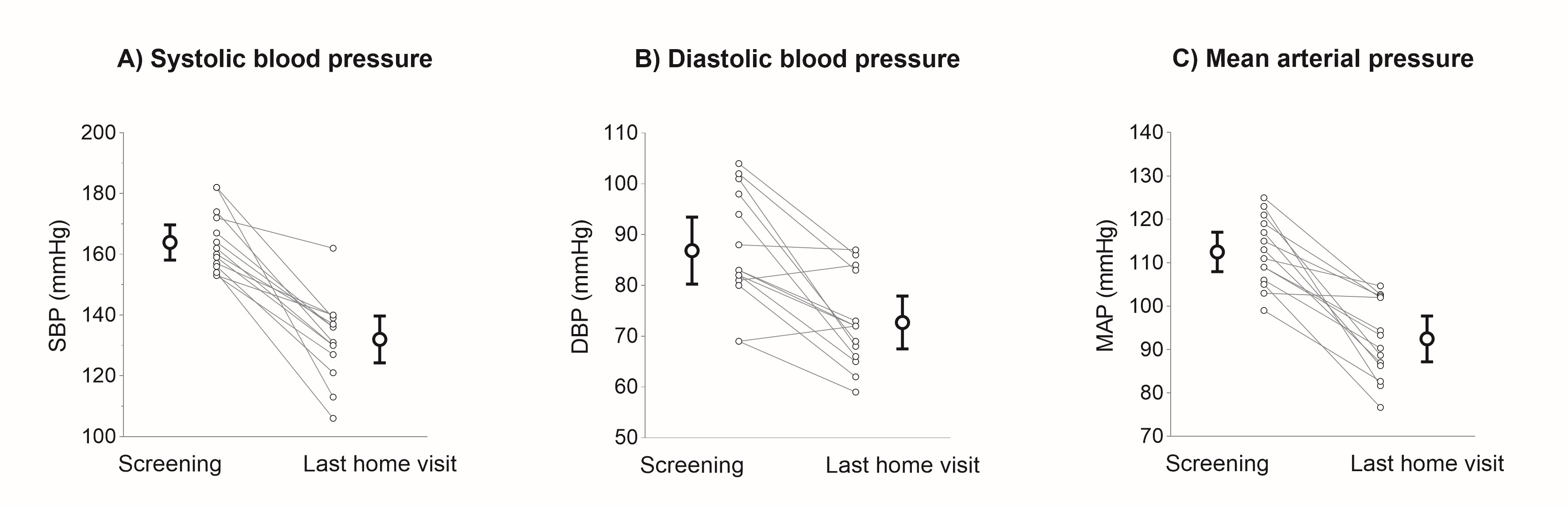


**Figure S2.** Graphs showing means with 95% confidence intervals for unattended systolic (A) and diastolic (B) blood pressure, and mean arterial pressure (C), assessed during the screening and last home visit, together with connected individual values.


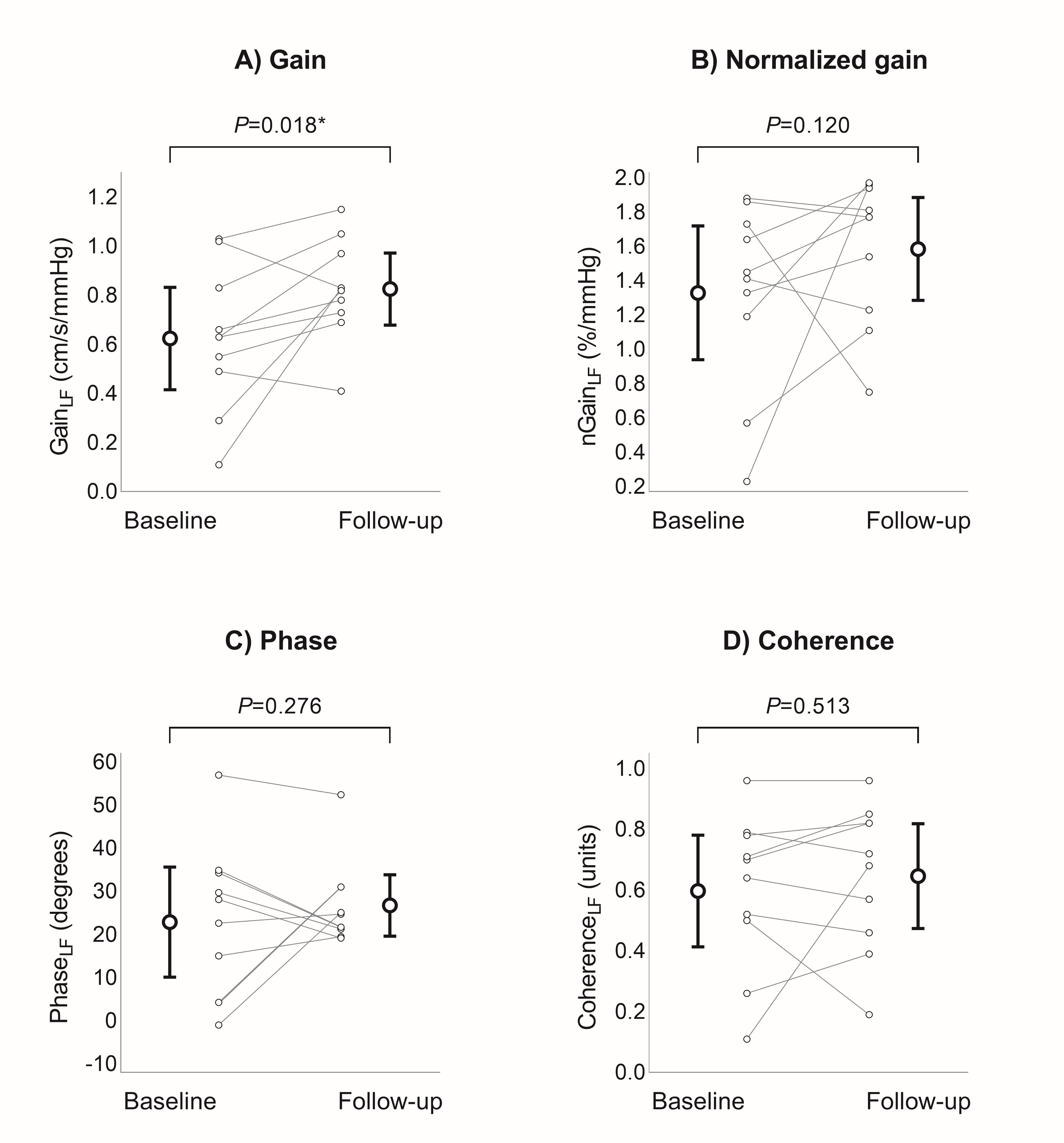


**Figure S3.** Graphs showing means with 95% confidence intervals for gain (A), normalized gain (B), phase (C), and coherence (D) over the low frequency domain assessed during baseline and following antihypertensive treatment, together with connected individual values.


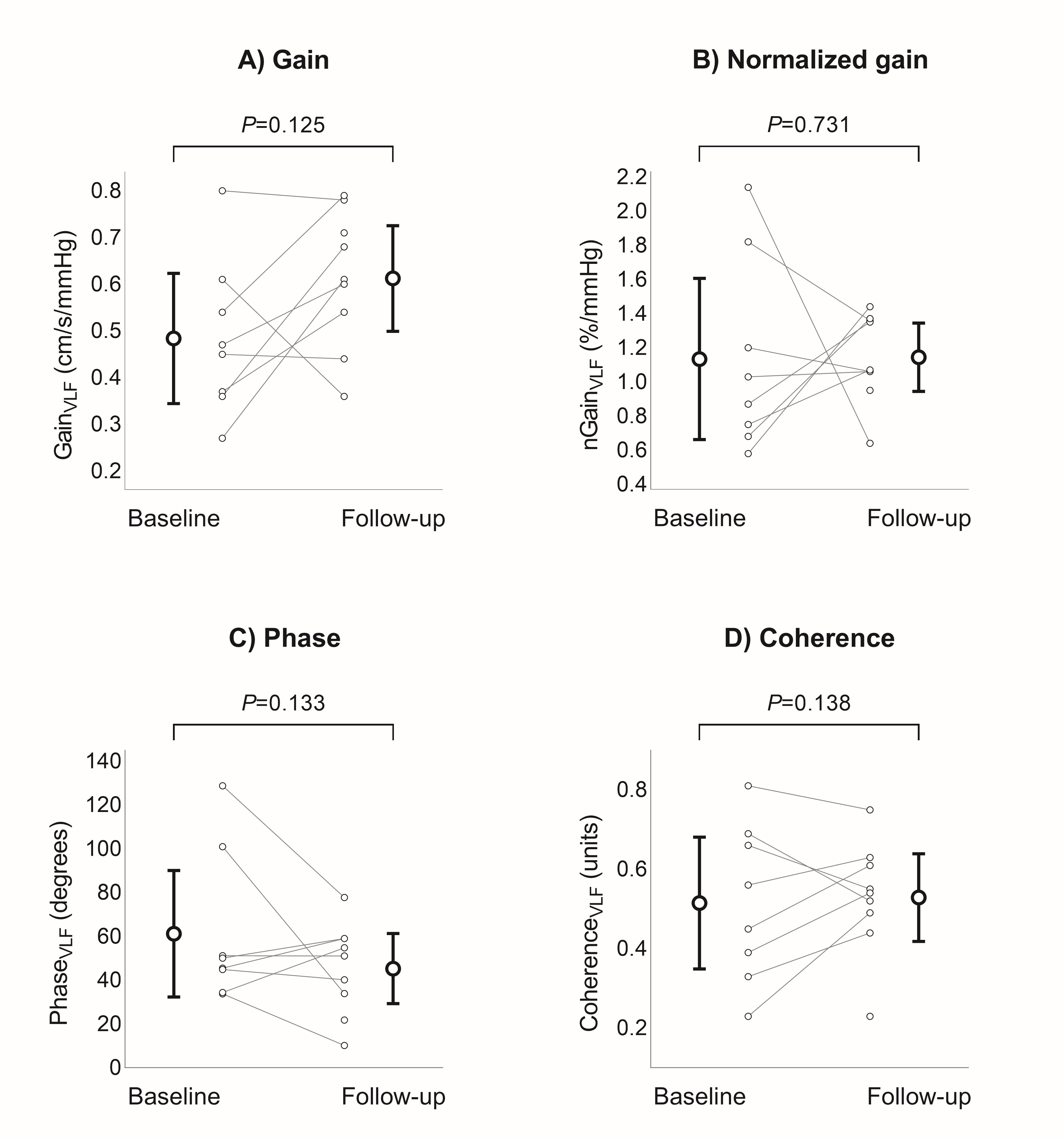


**Figure S4.** Graphs showing means with 95% confidence intervals for gain (A), normalized gain (B), phase (C), and coherence (D) over the very low frequency domain assessed during baseline and following antihypertensive treatment, together with connected individual values.
